# Comment on “A promising prognostic grading system incorporating weight loss and inflammation in patients with advanced cancer” by Zhang et al.

**DOI:** 10.1002/jcsm.13452

**Published:** 2024-03-11

**Authors:** Josh McGovern, Donald C. McMillan

**Affiliations:** ^1^ Academic Unit of Surgery, School of Medicine University of Glasgow Glasgow UK

Dear Editor,

We read the manuscript ‘A promising prognostic grading system incorporating weight loss and inflammation in patients with advanced cancer’ by Zhang and co‐workers with considerable interest. The authors are to be congratulated for carrying out a large multicentre prospective cohort study of 11 423 patients with advanced cancer that examined the prognostic value of the combination of weight loss and systemic inflammation, termed the weight loss and inflammation grading system (WLAIGS).^1^ The authors report that this combination captured the nutritional and inflammatory status of the host and was a robust predictor of survival. These results confirm the importance of the current consensus from the Global Leadership Initiative on Malnutrition (GLIM) that cancer cachexia is chronic disease‐related malnutrition with inflammation.^2^

It was of interest to note that the prevalence of systemic inflammation (neutrophil–lymphocyte ratio [NLR] > 3) was greater than that of weight loss (>2.5%) across disease stages (tumour–node–metastasis [TNM] stage III disease: 34% vs. 30%, respectively, and TNM stage IV disease: 54% vs. 37%, respectively). Moreover, NLR contributed more prognostic value in patients without weight loss compared with the prognostic value of weight loss in patients who were not systemically inflamed. These results have implications for how we should consider the currently proposed GLIM criteria and their definition of cancer cachexia.^2^ Specifically, systemic inflammation may dominate the prognostic value of body composition for survival in patients with advanced cancer. Furthermore, we are consistent with the concept that cachexia may be more aptly considered disease‐related inflammation with weight loss in the GLIM paradigm.^3^

Since the recognition of the importance of weight loss as a prognostic factor in ancient times, it has been a constant reference point in clinical diagnosis, prognosis and the treatment of cancer. More recently, with the recognition of systemic inflammation as an important prognostic factor, there has been increasing interest in incorporating a measure of systemic inflammation into routine clinical practice. To date, two measures have dominated the literature: the NLR and the modified Glasgow prognostic score (mGPS). However, although clinically routinely measured, compared with mGPS, the NLR is less sensitive to the presence of systemic inflammation, and the thresholds are not well defined.^4^ Given the limitations of the NLR and in the context of cancer immunotherapy, mGPS has been increasingly adopted for routine monitoring of the systemic inflammatory response.^5^, ^6^ Therefore, it would be of considerable interest to compare the relative prognostic value of NLR and mGPS in the WLAIGS.

As the authors point out, there are difficulties in accurately defining weight loss in routine cancer practice, and it has been proposed that computed tomography (CT)‐derived skeletal muscle mass may provide a more reliable measure of tissue loss.^7^ Indeed, it may be that the combination of relative muscle mass and systemic inflammation would provide a more clinically useful scoring system as both components can be objectively measured. It is of interest that recent secondary analyses of clinical trial data have reported that systemic inflammation dominates the prognostic value of CT‐derived muscle mass for survival outcomes in patients with advanced cancer.^8^, ^9^ Therefore, in light of the present observations, a comparison of the relative prognostic value of weight loss and relative skeletal mass in the context of systemic inflammation would be informative.

We thank the authors for providing important clinical data to underpin the theoretical basis of the GLIM criteria for cancer cachexia and to anchor the role of systemic inflammation as the dominant prognostic factor when nutritional and inflammatory status are considered in patients with advanced cancer.


Yours sincerely,Josh McGovern and Donald C. McMillan
